# 1-(4-Bromo-2-fluoro­benz­yl)pyridinium bis­(2-thioxo-1,3-dithiole-4,5-dithiol­ato)nickelate(III)

**DOI:** 10.1107/S1600536810039334

**Published:** 2010-10-09

**Authors:** Peng Zhang, Kaihui Li, Chongzhen Mei

**Affiliations:** a Institute of Environmental and Municipal Engineering, North China University of Water Conservancy and Electric Power, Zhengzhou 450011, People’s Republic of China

## Abstract

The title compound, (C_12_H_10_BrFN)[Ni(C_3_S_5_)_2_], is an ion-pair complex consisting of *N*-(2-fluoro-4-bromo­benz­yl)pyridinium cations and [Ni(dmit)_2_]^−^ anions (dmit = 2-thioxo-1,3-dithiole-4,5-dithiol­ate). In the anion, the Ni^III^ ion exhibits a square-planar coordination involving four S atoms from two dmit ligands. In the crystal structure, weak S⋯S [3.474 (3), 3.478 (3) and 3.547 (3) Å] and S⋯π [S⋯centroid distances = 3.360 (3), 3.378 (2), 3.537 (2) and 3.681 (3) Å] inter­actions and C—H⋯F hydrogen bonds lead to a three-dimensional supra­molecular network.

## Related literature

For general background to the network topologies and applications of bis­(dithiol­ate)–metal complexes, see: Cassoux (1999 ▶). For the synthesis, structures and properties of related complexes containing dmit ligands, see: Akutagawa & Nakamura (2000 ▶); Li *et al.* (2006 ▶); Zang *et al.* (2006 ▶, 2009 ▶). For lone-pair⋯π inter­actions, see: Egli & Sarkhel (2007 ▶). For the synthesis, see: Wang *et al.* (1998 ▶).
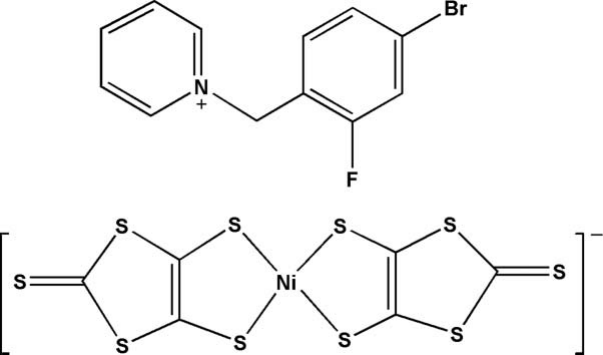


## Experimental

### 

#### Crystal data

(C_12_H_10_BrFN)[Ni(C_3_S_5_)_2_]
*M* *_r_* = 718.56
Triclinic, 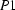

*a* = 6.2952 (15) Å
*b* = 9.716 (2) Å
*c* = 11.482 (3) Å
α = 65.953 (4)°
β = 77.592 (4)°
γ = 88.498 (4)°
*V* = 624.9 (3) Å^3^
*Z* = 1
Mo *K*α radiation
μ = 3.23 mm^−1^
*T* = 296 K
0.19 × 0.16 × 0.15 mm

#### Data collection

Bruker APEXII CCD diffractometer
Absorption correction: multi-scan (*SADABS*; Bruker, 2001 ▶) *T*_min_ = 0.579, *T*_max_ = 0.643
3111 measured reflections
2619 independent reflections
2434 reflections with *I* > 2σ(*I*)
*R*_int_ = 0.022

#### Refinement

*R*[*F* ^2^ > 2σ(*F* ^2^)] = 0.037
*wR*(*F* ^2^) = 0.086
*S* = 1.03
2619 reflections
290 parameters
3 restraints
H-atom parameters constrained
Δρ_max_ = 0.47 e Å^−3^
Δρ_min_ = −0.52 e Å^−3^
Absolute structure: Flack (1983 ▶); 456 Friedel pairs
Flack parameter: 0.364 (16)

### 

Data collection: *APEX2* (Bruker, 2007 ▶); cell refinement: *SAINT* (Bruker, 2007 ▶); data reduction: *SAINT*; program(s) used to solve structure: *SHELXS97* (Sheldrick, 2008 ▶); program(s) used to refine structure: *SHELXL97* (Sheldrick, 2008 ▶); molecular graphics: *DIAMOND* (Brandenburg, 1999 ▶); software used to prepare material for publication: *SHELXTL* (Sheldrick, 2008 ▶).

## Supplementary Material

Crystal structure: contains datablocks I, global. DOI: 10.1107/S1600536810039334/hy2356sup1.cif

Structure factors: contains datablocks I. DOI: 10.1107/S1600536810039334/hy2356Isup2.hkl

Additional supplementary materials:  crystallographic information; 3D view; checkCIF report

## Figures and Tables

**Table 1:** Selected bond lengths (Å)

Ni1—S4	2.163 (3)
Ni1—S5	2.150 (2)
Ni1—S6	2.157 (2)
Ni1—S7	2.169 (3)

**Table 2:** Hydrogen-bond geometry (Å, °)

*D*—H⋯*A*	*D*—H	H⋯*A*	*D*⋯*A*	*D*—H⋯*A*
C14—H14⋯F1^i^	0.93	2.60	3.476 (11)	156

Symmetry code: (i) 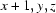.

**Fig. 1.**
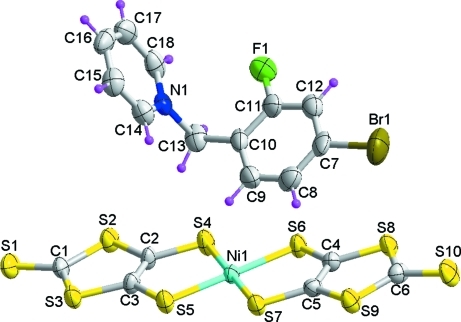
The structures of the cation and anion in the title compound, with displacement ellipsoids drawn at the 50% probability level.

**Fig. 2.**
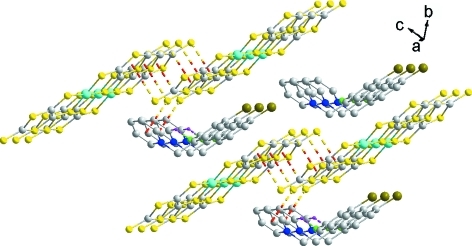
Three-dimensional supramolecular structure of the title compound. H atoms have been omitted for clarity. Dashed lines indicate weak S···S, S···π and C—H···F interactions.

## supplementary crystallographic information

### Comment

Extensive research has been focused on the synthesis and characterization of bis(dithiolate)–metal complexes and their analogues, due to their properties and potential applications as conducting, magnetic and non-linear optical (NLO) materials (Cassoux, 1999). 2-Thioxo-1,3-dithiole-4,5-dithiolate (dmit) metal complex also is excellent building block employed for the construction of molecular magnetic materials (Li *et al.*, 2006; Zang *et al.*, 2006, 2009) apart from its well known electric conductivity as molecular conductors (Akutagawa & Nakamura, 2000). We report herein the synthesis and crystal structure of the title compound, a new ion-pair complex.

The title compound comprises [Ni^III^(dmit)_2_]^-^ anions and *N*-(2-fluoro-4-bromobenzyl)pyridinium cations (Fig. 1). The Ni^III^ ion adopts a square-planar geometry coordinated by four S atoms from two dmit ligands, with Ni—S bond lengths ranging from 2.150 (2) to 2.169 (3) Å (Table 1). The [Ni^III^(dmit)_2_]^-^ anions are in a parallel arrangement, with S···S interactions ranging from 3.474 (3) to 3.547 (3) Å. Two neighbouring anions are parallel in a head-to-tail inversion arrangement so that lone-pair (lp)···π (Egli & Sarkhel, 2007) interactions form between one terminal S atom of the anion and the other terminal π system of adjacent anion [S1···Cg1^i^ = 3.378 (2) and S10^i^···Cg2 = 3.537 (2) Å. Cg1 and Cg2 are the centroids of C4–C6, S8, S9 ring and C1–C3, S2, S3 ring, respectively. Symmetry code: (i) x, -1+y, 1+z]. The anion and the neighbouring cation are also associated together through lp···π interactions between two terminal S atoms of the anion and the pyridine rings of two different cations [S1···Cg3^ii^ = 3.360 (3) and S10···Cg3^iii^ = 3.681 (3) Å. Cg3 is the centroid of C14–C18, N1 ring. Symmetry codes: (ii) 1+x, -1+y, z; (iii) x, 1+y, -1+z]. The weak S···S and S(lp)···π interactions lead to a three-dimensional supramolecular structure. In addition, the cations adopt a parallel arrangement, and the shortest distance between H14 from the pyridine ring of a cation and F1 atom from the neighbouring cation is 2.60 Å, indicating the existence of a C—H···F hydrogen bond (Table 2), which stabilizes the three-dimensional structure (Fig. 2).

### Experimental

4,5-Bis(thiobenzoyl)-1,3-dithiole-2-thione (812 mg, 2.0 mmol) (Wang *et al.*, 1998) was suspended in dry methanol (20 ml) and sodium (92 mg, 4.0 mmol) was added under a nitrogen atmosphere at room temperature to give a bright-red solution. NiCl_2_.6H_2_O (238 mg, 1 mmol) was then added, followed successively by addition of I_2_ (127 mg, 0.5 mmol) and a solution of *N*-(2-fluoro-4-bromobenzyl)pyridinium bromide (346 mg, 1 mmol) in methanol at an interval of approximately 20 min. The solution was stirred for a further 30 min and the resulting solid was collected by filtration. Single crystals of the title compound were obtained by evaporation of a dilute acetone solution over 1–2 weeks at room temperature.

### Refinement

H atoms were positioned geometrically and refined using a riding model, with C—H = 0.93(aromatic) and 0.97(CH_2_) Å and *U*_iso_(H) = 1.2*U*_eq_(C).

### Crystal data

**Table:**

(C_12_H_10_BrFN)[Ni(C_3_S_5_)_2_]	*Z* = 1
*M**_r_* = 718.56	*F*(000) = 357
Triclinic, *P*1	*D*_x_ = 1.909 Mg m^−^^3^
Hall symbol: P 1	Mo *K*α radiation, λ = 0.71073 Å
*a* = 6.2952 (15) Å	Cell parameters from 617 reflections
*b* = 9.716 (2) Å	θ = 3.5–25.2°
*c* = 11.482 (3) Å	µ = 3.23 mm^−^^1^
α = 65.953 (4)°	*T* = 296 K
β = 77.592 (4)°	Block, black
γ = 88.498 (4)°	0.19 × 0.16 × 0.15 mm
*V* = 624.9 (3) Å^3^	

### Data collection

**Table:**

Bruker APEXII CCD diffractometer	2619 independent reflections
Radiation source: fine-focus sealed tube	2434 reflections with *I* > 2σ(*I*)
graphite	*R*_int_ = 0.022
φ and ω scans	θ_max_ = 25.0°, θ_min_ = 2.0°
Absorption correction: multi-scan (*SADABS*; Bruker, 2001)	*h* = −6→7
*T*_min_ = 0.579, *T*_max_ = 0.643	*k* = −11→11
3111 measured reflections	*l* = −13→12

### Refinement

**Table:**

Refinement on *F*^2^	Secondary atom site location: difference Fourier map
Least-squares matrix: full	Hydrogen site location: inferred from neighbouring sites
*R*[*F*^2^ > 2σ(*F*^2^)] = 0.037	H-atom parameters constrained
*wR*(*F*^2^) = 0.086	*w* = 1/[σ^2^(*F*_o_^2^) + (0.0398*P*)^2^] where *P* = (*F*_o_^2^ + 2*F*_c_^2^)/3
*S* = 1.03	(Δ/σ)_max_ < 0.001
2619 reflections	Δρ_max_ = 0.47 e Å^−^^3^
290 parameters	Δρ_min_ = −0.52 e Å^−^^3^
3 restraints	Absolute structure: Flack (1983); 456 Friedel pairs
Primary atom site location: structure-invariant direct methods	Flack parameter: 0.364 (16)

### Fractional atomic coordinates and isotropic or equivalent isotropic displacement parameters (Å^2^)

**Table:**

	*x*	*y*	*z*	*U*_iso_*/*U*_eq_	
Ni1	0.82342 (18)	0.80439 (11)	0.41205 (10)	0.0318 (2)	
C1	0.9321 (14)	0.2595 (8)	0.7954 (7)	0.039 (2)	
C2	0.7858 (16)	0.4827 (8)	0.6172 (7)	0.037 (2)	
C3	0.9769 (16)	0.5382 (9)	0.6182 (8)	0.036 (2)	
C4	0.6622 (16)	1.0780 (9)	0.2125 (7)	0.036 (2)	
C5	0.8601 (15)	1.1313 (9)	0.2140 (8)	0.035 (2)	
C6	0.7024 (15)	1.3601 (8)	0.0500 (8)	0.040 (2)	
C7	0.3261 (16)	1.2596 (10)	0.4477 (7)	0.055 (2)	
C8	0.5284 (16)	1.2057 (10)	0.4587 (8)	0.057 (2)	
H8	0.6539	1.2656	0.4052	0.068*	
C9	0.5428 (14)	1.0646 (10)	0.5484 (8)	0.053 (2)	
H9	0.6802	1.0288	0.5548	0.064*	
C10	0.3607 (14)	0.9712 (9)	0.6311 (7)	0.046 (2)	
C11	0.1592 (13)	1.0304 (9)	0.6158 (8)	0.0470 (19)	
C12	0.1398 (16)	1.1716 (10)	0.5272 (8)	0.053 (2)	
H12	0.0031	1.2083	0.5203	0.063*	
C13	0.3711 (18)	0.8139 (10)	0.7295 (9)	0.053 (2)	
H13A	0.2670	0.7469	0.7230	0.064*	
H13B	0.5155	0.7800	0.7103	0.064*	
C14	0.4625 (15)	0.8705 (9)	0.9011 (8)	0.049 (2)	
H14	0.5909	0.9200	0.8419	0.059*	
C15	0.4212 (16)	0.8663 (9)	1.0238 (9)	0.054 (2)	
H15	0.5214	0.9105	1.0493	0.065*	
C16	0.229 (2)	0.7954 (11)	1.1092 (10)	0.063 (3)	
H16	0.1965	0.7928	1.1930	0.076*	
C17	0.0853 (17)	0.7290 (10)	1.0716 (10)	0.067 (3)	
H17	−0.0453	0.6812	1.1292	0.080*	
C18	0.1351 (16)	0.7331 (9)	0.9473 (9)	0.055 (2)	
H18	0.0395	0.6862	0.9210	0.066*	
S1	0.9660 (4)	0.0969 (2)	0.9092 (2)	0.0527 (6)	
S2	0.7008 (4)	0.2974 (2)	0.7299 (2)	0.0454 (6)	
S3	1.1216 (4)	0.4113 (2)	0.7263 (2)	0.0470 (6)	
S4	0.6256 (4)	0.5934 (2)	0.5149 (2)	0.0457 (6)	
S5	1.0756 (4)	0.7183 (2)	0.5178 (2)	0.0418 (5)	
S6	0.5691 (4)	0.8923 (2)	0.30748 (19)	0.0407 (5)	
S7	1.0202 (3)	1.0169 (2)	0.31248 (19)	0.0386 (5)	
S8	0.5130 (3)	1.2069 (2)	0.11229 (19)	0.0412 (5)	
S9	0.9310 (3)	1.3204 (2)	0.1136 (2)	0.0447 (6)	
S10	0.6629 (4)	1.5263 (2)	−0.0602 (2)	0.0577 (6)	
N1	0.3225 (10)	0.8051 (6)	0.8643 (6)	0.0399 (14)	
F1	−0.0228 (8)	0.9428 (6)	0.6944 (5)	0.0695 (14)	
Br1	0.3003 (3)	1.45136 (15)	0.31995 (14)	0.0902 (4)	

### Atomic displacement parameters (Å^2^)

**Table:**

	*U*^11^	*U*^22^	*U*^33^	*U*^12^	*U*^13^	*U*^23^
Ni1	0.0314 (4)	0.0287 (4)	0.0289 (4)	0.0020 (3)	−0.0081 (3)	−0.0049 (3)
C1	0.041 (5)	0.034 (4)	0.038 (4)	0.012 (4)	−0.012 (4)	−0.009 (4)
C2	0.043 (6)	0.027 (4)	0.029 (4)	0.000 (4)	−0.012 (4)	0.003 (3)
C3	0.043 (6)	0.028 (4)	0.031 (4)	0.002 (4)	−0.011 (4)	−0.004 (4)
C4	0.035 (6)	0.034 (5)	0.032 (4)	0.006 (4)	−0.006 (4)	−0.008 (4)
C5	0.032 (5)	0.031 (4)	0.038 (4)	0.003 (4)	−0.005 (4)	−0.012 (4)
C6	0.047 (6)	0.025 (4)	0.039 (5)	0.002 (4)	−0.002 (4)	−0.009 (3)
C7	0.080 (7)	0.068 (6)	0.026 (5)	0.028 (6)	−0.021 (5)	−0.024 (4)
C8	0.060 (6)	0.069 (6)	0.041 (5)	−0.001 (5)	−0.013 (4)	−0.020 (5)
C9	0.047 (5)	0.070 (6)	0.048 (5)	0.018 (4)	−0.020 (4)	−0.026 (5)
C10	0.061 (5)	0.050 (5)	0.035 (4)	0.025 (4)	−0.022 (4)	−0.020 (4)
C11	0.048 (5)	0.059 (5)	0.039 (4)	0.004 (4)	−0.015 (4)	−0.022 (4)
C12	0.063 (6)	0.062 (5)	0.046 (5)	0.022 (4)	−0.024 (4)	−0.029 (4)
C13	0.072 (7)	0.050 (5)	0.057 (5)	0.015 (5)	−0.032 (5)	−0.033 (5)
C14	0.048 (5)	0.041 (4)	0.061 (5)	0.007 (4)	−0.022 (4)	−0.019 (4)
C15	0.072 (6)	0.046 (5)	0.057 (6)	0.010 (4)	−0.028 (5)	−0.027 (4)
C16	0.092 (9)	0.048 (6)	0.037 (5)	0.027 (6)	−0.008 (5)	−0.010 (4)
C17	0.055 (6)	0.052 (5)	0.063 (7)	0.008 (4)	−0.004 (5)	0.002 (5)
C18	0.055 (6)	0.029 (4)	0.071 (6)	0.002 (4)	−0.026 (5)	−0.006 (4)
S1	0.0627 (17)	0.0352 (11)	0.0467 (12)	0.0142 (10)	−0.0127 (11)	−0.0037 (9)
S2	0.0468 (15)	0.0308 (11)	0.0462 (12)	−0.0004 (9)	−0.0130 (10)	−0.0021 (9)
S3	0.0410 (15)	0.0434 (12)	0.0439 (12)	0.0082 (10)	−0.0157 (10)	−0.0027 (10)
S4	0.0414 (15)	0.0337 (11)	0.0515 (13)	−0.0021 (9)	−0.0221 (11)	−0.0010 (9)
S5	0.0361 (14)	0.0358 (11)	0.0437 (12)	−0.0010 (9)	−0.0170 (10)	−0.0025 (9)
S6	0.0377 (14)	0.0321 (11)	0.0419 (12)	−0.0024 (9)	−0.0169 (10)	−0.0006 (9)
S7	0.0319 (13)	0.0338 (10)	0.0416 (11)	0.0001 (9)	−0.0131 (9)	−0.0043 (9)
S8	0.0336 (14)	0.0343 (10)	0.0454 (12)	0.0032 (9)	−0.0144 (10)	−0.0035 (9)
S9	0.0417 (14)	0.0301 (11)	0.0534 (13)	0.0006 (9)	−0.0154 (10)	−0.0060 (9)
S10	0.0582 (16)	0.0325 (11)	0.0651 (14)	0.0106 (11)	−0.0203 (12)	−0.0001 (10)
N1	0.045 (4)	0.035 (3)	0.045 (4)	0.015 (3)	−0.020 (3)	−0.017 (3)
F1	0.054 (3)	0.080 (4)	0.071 (3)	0.004 (3)	−0.016 (3)	−0.027 (3)
Br1	0.1427 (12)	0.0616 (6)	0.0614 (6)	0.0258 (6)	−0.0418 (7)	−0.0123 (5)

### Geometric parameters (Å, °)

**Table:**

Ni1—S4	2.163 (3)	C8—C9	1.353 (12)
Ni1—S5	2.150 (2)	C8—H8	0.9300
Ni1—S6	2.157 (2)	C9—C10	1.387 (13)
Ni1—S7	2.169 (3)	C9—H9	0.9300
C1—S1	1.634 (7)	C10—C11	1.395 (11)
C1—S3	1.720 (9)	C10—C13	1.496 (11)
C1—S2	1.743 (8)	C11—F1	1.354 (10)
C2—C3	1.335 (12)	C11—C12	1.356 (11)
C2—S4	1.725 (8)	C12—H12	0.9300
C2—S2	1.748 (8)	C13—N1	1.479 (10)
C3—S5	1.698 (8)	C13—H13A	0.9700
C3—S3	1.752 (8)	C13—H13B	0.9700
C4—C5	1.368 (12)	C14—N1	1.329 (10)
C4—S6	1.722 (9)	C14—C15	1.360 (11)
C4—S8	1.733 (8)	C14—H14	0.9300
C5—S7	1.714 (8)	C15—C16	1.374 (14)
C5—S9	1.732 (8)	C15—H15	0.9300
C6—S10	1.649 (7)	C16—C17	1.357 (15)
C6—S9	1.716 (9)	C16—H16	0.9300
C6—S8	1.734 (9)	C17—C18	1.378 (13)
C7—C8	1.376 (12)	C17—H17	0.9300
C7—C12	1.377 (14)	C18—N1	1.344 (11)
C7—Br1	1.873 (9)	C18—H18	0.9300
			
S5—Ni1—S6	179.27 (12)	F1—C11—C10	118.0 (8)
S5—Ni1—S4	92.78 (9)	C12—C11—C10	122.6 (9)
S6—Ni1—S4	87.41 (10)	C11—C12—C7	118.8 (8)
S5—Ni1—S7	86.86 (10)	C11—C12—H12	120.6
S6—Ni1—S7	92.94 (8)	C7—C12—H12	120.6
S4—Ni1—S7	178.83 (12)	N1—C13—C10	111.6 (6)
S1—C1—S3	123.7 (5)	N1—C13—H13A	109.3
S1—C1—S2	123.6 (6)	C10—C13—H13A	109.3
S3—C1—S2	112.7 (4)	N1—C13—H13B	109.3
C3—C2—S4	121.0 (6)	C10—C13—H13B	109.3
C3—C2—S2	116.9 (6)	H13A—C13—H13B	108.0
S4—C2—S2	122.1 (6)	N1—C14—C15	121.2 (9)
C2—C3—S5	122.0 (6)	N1—C14—H14	119.4
C2—C3—S3	115.6 (6)	C15—C14—H14	119.4
S5—C3—S3	122.4 (6)	C14—C15—C16	118.7 (9)
C5—C4—S6	120.8 (6)	C14—C15—H15	120.7
C5—C4—S8	116.5 (6)	C16—C15—H15	120.7
S6—C4—S8	122.7 (6)	C17—C16—C15	120.2 (10)
C4—C5—S7	121.2 (6)	C17—C16—H16	119.9
C4—C5—S9	115.4 (6)	C15—C16—H16	119.9
S7—C5—S9	123.4 (6)	C16—C17—C18	119.4 (10)
S10—C6—S9	124.2 (5)	C16—C17—H17	120.3
S10—C6—S8	122.3 (5)	C18—C17—H17	120.3
S9—C6—S8	113.5 (4)	N1—C18—C17	119.6 (9)
C8—C7—C12	120.6 (8)	N1—C18—H18	120.2
C8—C7—Br1	120.3 (8)	C17—C18—H18	120.2
C12—C7—Br1	119.1 (7)	C1—S2—C2	96.9 (4)
C9—C8—C7	119.3 (9)	C1—S3—C3	97.9 (4)
C9—C8—H8	120.4	C2—S4—Ni1	101.8 (3)
C7—C8—H8	120.4	C3—S5—Ni1	102.5 (3)
C8—C9—C10	122.6 (8)	C4—S6—Ni1	102.6 (3)
C8—C9—H9	118.7	C5—S7—Ni1	102.4 (3)
C10—C9—H9	118.7	C4—S8—C6	96.8 (4)
C9—C10—C11	116.1 (7)	C6—S9—C5	97.8 (4)
C9—C10—C13	123.8 (8)	C14—N1—C18	120.9 (7)
C11—C10—C13	120.1 (9)	C14—N1—C13	119.7 (8)
F1—C11—C12	119.4 (7)	C18—N1—C13	119.4 (8)
			
S4—C2—C3—S5	−1.1 (11)	C2—C3—S3—C1	−2.7 (8)
S2—C2—C3—S5	−177.9 (5)	S5—C3—S3—C1	179.0 (5)
S4—C2—C3—S3	−179.4 (5)	C3—C2—S4—Ni1	1.2 (8)
S2—C2—C3—S3	3.8 (9)	S2—C2—S4—Ni1	177.8 (5)
S6—C4—C5—S7	−1.2 (10)	S5—Ni1—S4—C2	−0.7 (3)
S8—C4—C5—S7	178.1 (4)	S6—Ni1—S4—C2	−180.0 (3)
S6—C4—C5—S9	−179.9 (4)	C2—C3—S5—Ni1	0.4 (8)
S8—C4—C5—S9	−0.5 (9)	S3—C3—S5—Ni1	178.6 (5)
C12—C7—C8—C9	0.3 (12)	S4—Ni1—S5—C3	0.3 (3)
Br1—C7—C8—C9	−177.5 (6)	S7—Ni1—S5—C3	179.2 (3)
C7—C8—C9—C10	−0.5 (12)	C5—C4—S6—Ni1	2.5 (7)
C8—C9—C10—C11	0.8 (12)	S8—C4—S6—Ni1	−176.8 (4)
C8—C9—C10—C13	178.9 (8)	S4—Ni1—S6—C4	176.6 (3)
C9—C10—C11—F1	179.8 (7)	S7—Ni1—S6—C4	−2.2 (3)
C13—C10—C11—F1	1.7 (11)	C4—C5—S7—Ni1	−0.7 (7)
C9—C10—C11—C12	−1.1 (11)	S9—C5—S7—Ni1	177.8 (4)
C13—C10—C11—C12	−179.2 (7)	S5—Ni1—S7—C5	−177.5 (3)
F1—C11—C12—C7	−179.9 (7)	S6—Ni1—S7—C5	1.8 (3)
C10—C11—C12—C7	1.0 (11)	C5—C4—S8—C6	1.3 (7)
C8—C7—C12—C11	−0.6 (12)	S6—C4—S8—C6	−179.4 (5)
Br1—C7—C12—C11	177.3 (5)	S10—C6—S8—C4	178.1 (5)
C9—C10—C13—N1	106.1 (9)	S9—C6—S8—C4	−1.6 (5)
C11—C10—C13—N1	−75.9 (10)	S10—C6—S9—C5	−178.3 (5)
N1—C14—C15—C16	−1.3 (12)	S8—C6—S9—C5	1.4 (5)
C14—C15—C16—C17	1.1 (13)	C4—C5—S9—C6	−0.5 (7)
C15—C16—C17—C18	0.2 (13)	S7—C5—S9—C6	−179.1 (5)
C16—C17—C18—N1	−1.3 (12)	C15—C14—N1—C18	0.2 (11)
S1—C1—S2—C2	−178.6 (5)	C15—C14—N1—C13	179.1 (7)
S3—C1—S2—C2	0.9 (5)	C17—C18—N1—C14	1.1 (11)
C3—C2—S2—C1	−2.9 (8)	C17—C18—N1—C13	−177.8 (7)
S4—C2—S2—C1	−179.6 (5)	C10—C13—N1—C14	−68.7 (10)
S1—C1—S3—C3	−179.8 (5)	C10—C13—N1—C18	110.3 (9)
S2—C1—S3—C3	0.7 (6)		

### Hydrogen-bond geometry (Å, °)

**Table:**

*D*—H···*A*	*D*—H	H···*A*	*D*···*A*	*D*—H···*A*
C14—H14···F1^i^	0.93	2.60	3.476 (11)	156

Symmetry codes: (i) *x*+1, *y*, *z*.
